# *In Situ* Activation and Heterologous Production of a Cryptic Lantibiotic from an African Plant Ant-Derived *Saccharopolyspora* Species

**DOI:** 10.1128/AEM.01876-19

**Published:** 2020-01-21

**Authors:** Eleni Vikeli, David A. Widdick, Sibyl F. D. Batey, Daniel Heine, Neil A. Holmes, Mervyn J. Bibb, Dino J. Martins, Naomi E. Pierce, Matthew I. Hutchings, Barrie Wilkinson

**Affiliations:** a Department of Molecular Microbiology, John Innes Centre, Norwich, United Kingdom; b School of Biological Sciences, University of East Anglia, Norwich, United Kingdom; c Department of Organismic and Evolutionary Biology, Harvard University, Cambridge, Massachusetts, USA; d Mpala Research Centre, Nanyuki, Kenya; INRS—Institut Armand-Frappier

**Keywords:** lantibiotic, lanthipeptide, plant-ant, *Saccharopolyspora*, cinnamycin

## Abstract

Most clinical antibiotics are derived from actinomycete natural products discovered at least 60 years ago. However, the repeated rediscovery of known compounds led the pharmaceutical industry to largely discard microbial natural products (NPs) as a source of new chemical diversity. Recent advances in genome sequencing have revealed that these organisms have the potential to make many more NPs than previously thought. Approaches to unlock NP biosynthesis by genetic manipulation of strains, by the application of chemical genetics, or by microbial cocultivation have resulted in the identification of new antibacterial compounds. Concomitantly, intensive exploration of coevolved ecological niches, such as insect-microbe defensive symbioses, has revealed these to be a rich source of chemical novelty. Here, we report the new lanthipeptide antibiotic kyamicin, which was generated through the activation of a cryptic biosynthetic gene cluster identified by genome mining *Saccharopolyspora* species found in the obligate domatium-dwelling ant Tetraponera penzigi of the ant plant Vachellia drepanolobium. Transcriptional activation of this silent gene cluster was achieved by ectopic expression of a pathway-specific activator under the control of a constitutive promoter. Subsequently, a heterologous production platform was developed which enabled the purification of kyamicin for structural characterization and bioactivity determination. This strategy was also successful for the production of lantibiotics from other genera, paving the way for a synthetic heterologous expression platform for the discovery of lanthipeptides that are not detected under laboratory conditions or that are new to nature.

**IMPORTANCE** The discovery of novel antibiotics to tackle the growing threat of antimicrobial resistance is impeded by difficulties in accessing the full biosynthetic potential of microorganisms. The development of new tools to unlock the biosynthesis of cryptic bacterial natural products will greatly increase the repertoire of natural product scaffolds. Here, we report a strategy for the ectopic expression of pathway-specific positive regulators that can be rapidly applied to activate the biosynthesis of cryptic lanthipeptide biosynthetic gene clusters. This allowed the discovery of a new lanthipeptide antibiotic directly from the native host and via heterologous expression.

## Abstract (precis)

The discovery of novel antibiotics to tackle the growing threat of antimicrobial resistance is impeded by difficulties in accessing the full biosynthetic potential of microorganisms. The development of new tools to unlock the biosynthesis of cryptic bacterial natural products will greatly increase the repertoire of natural product scaffolds. Here, we report a strategy for the ectopic expression of pathway-specific positive regulators that can be rapidly applied to activate the biosynthesis of cryptic lanthipeptide biosynthetic gene clusters. This allowed the discovery of a new lanthipeptide antibiotic directly from the native host and via heterologous expression.

## INTRODUCTION

Antimicrobial resistance (AMR) is arguably the greatest health threat facing humanity in the 21st century (1–3). It is predicted that without urgent action, infectious disease will become the biggest killer of humans by 2050 (1). The majority of clinically used antibiotics are based on microbial natural products, isolated mostly from soil-dwelling *Streptomyces* species and other filamentous actinomycete bacteria, and these organisms remain a promising source of new antibiotics. Although the discovery pipeline began to dry up in the 1960s, blighted by the rediscovery of known compounds, we know from large-scale genome sequencing that up to 90% of microbial natural products are not produced under laboratory conditions (4). Thus, there exists a wealth of novel chemistry waiting to be discovered by mining the genomes of these organisms. Bearing in mind that >600 *Streptomyces* species and many other so-called “rare” actinomycetes have been described, thousands of potentially useful but “cryptic” bioactive compounds are waiting to be discovered, even from well-characterized strains (5, 6). Several approaches have been taken to activate cryptic pathways, including the heterologous expression of entire biosynthetic gene clusters (BGCs) in optimized *Streptomyces* host strains and the rewiring of BGCs to bypass their natural regulatory mechanisms (7). The knowledge that we have barely sampled the biosynthetic capabilities of known strains, and that even well-explored environments such as soil have been undersampled for antibiotic-producing microbes, provides a much-needed opportunity for the development of new natural product-based antibiotics.

Searching symbiotic niches for new actinomycete strains also shows great promise for discovering new natural products (8–11). We previously described the formicamycins, new polyketides with potent Gram-positive antibacterial activity produced by a new *Streptomyces* species that we named Streptomyces formicae KY5 (12). This species was isolated from a phytoecious ant species, Tetraponera penzigi, whose colonies inhabit the African ant plant *Vachellia* (*Acacia*) *drepanolobium*. The ants were collected in Kenya, hence the KY strain designation (13). These ants live in symbiosis with their host plants, the “whistling thorn acacias,” that have evolved specialized hollow stipular thorns called domatia to house the ants (14). In return for housing, plant ants protect their hosts against attack by large herbivores, including elephants (15), and recent reports have suggested that they grow specialized fungal communities inside their domatia, possibly as a food source for their larvae (16, 17). The external cuticular microbiome of *T. penzigi* ants is heterogeneous, and unbiased methods have shown that it is dominated by members of the phyla *Proteobacteria* and *Firmicutes*, with *Actinobacteria* forming a minor component (13). This contrasts with the better-studied fungus-farming leafcutter ants of the tribe Attini, which are dominated by actinobacteria, specifically, by a single strain of *Pseudonocardia* that can be vertically transmitted by the new queens (18, 19). Leafcutter ants feed cut plant material to their symbiotic food fungus Leucoagaricus gongylophorus and use antifungals made by their *Pseudonocardia* symbionts to defend their food fungus against fungal parasites in the genus *Escovopsis* (20–22). Despite the low abundance of *Actinobacteria*, we isolated several strains, including three from the rare actinomycete genus *Saccharopolyspora*, which, despite the modest number of described species, is the origin of the medically and agriculturally important natural products erythromycin and spinosyn. Erythromycin is a well-established clinical antibiotic that inhibits protein synthesis through binding to the 50S subunit of the ribosome (23). The spinosyns are structurally unique insecticides used for the control of insect pests and the protection of grain products. They derive from the fermentation of Saccharopolyspora spinosa and have potent activity and low environmental effect (24).

**FIG 1:**
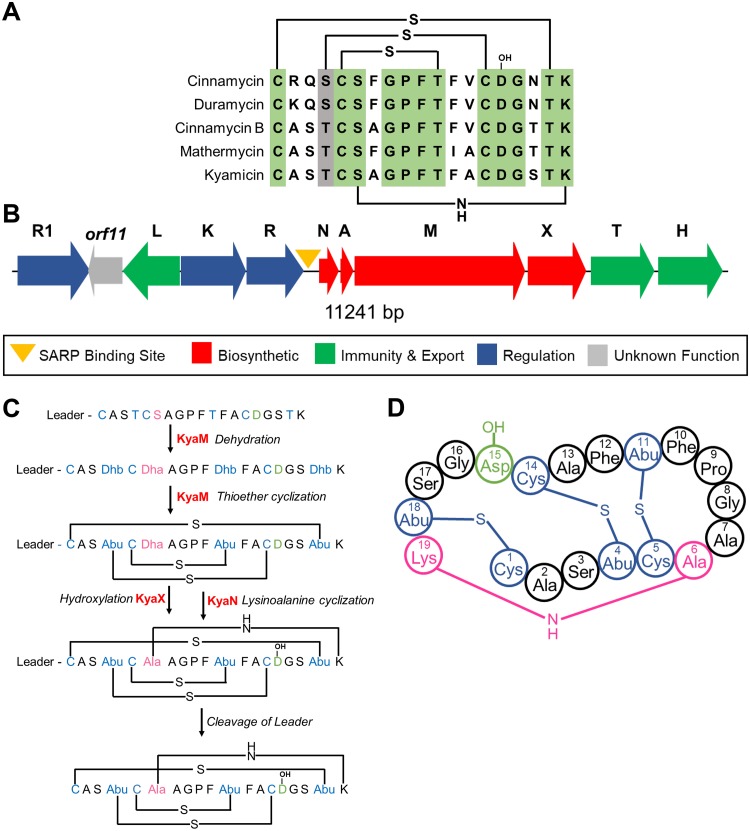
Kyamicin peptide sequence and biosynthesis. (A) Alignment of core peptides of kyamicin and a selection of known type B cinnamycin-like lantibiotics, with the positions of the thioether and lysinoalanine bridges in the mature peptide shown. Conserved residues are highlighted in green, and similar residues are highlighted in gray. (B) The kyamicin biosynthetic gene cluster, with genes colored according to predicted function. (C) Schematic of kyamicin biosynthesis. The thioether bridges are formed first by dehydration of Thr4, Thr11, Thr18, and Ser6 by KyaM to form dehydrobutyrine (Dhb) and dehydroalanine (Dha) residues, respectively. After thioether cyclization by KyaM, Dhb becomes S-linked aminobutyric acid (Abu) and Dha becomes S-linked Ala. Asp15 is hydroxylated by KyaX, and the lysinoalanine bridge is then formed between Dha6 and Lys19 by KyaN. After the core peptide is fully modified, the leader peptide is proteolytically cleaved. (D) Structural representation of the mature kyamicin lantibiotic.

Genome mining of the isolated *Saccharopolyspora* strains identified a conserved BGC encoding a putative cinnamycin-like lanthipeptide antibiotic (lantibiotic) (25), although no products for this BGC could be identified from the wild-type isolates. Cinnamycin is a class II type B lantibiotic produced by Streptomyces cinnamoneus DSM 40005 which destabilizes the cytoplasmic membrane by binding phosphatidylethanolamine (PE) (25–27). Lanthipeptides belong to the ribosomally synthesized and posttranslationally modified peptide (RiPP) family of natural products (28, 29), and cinnamycin is the founding member of a subgroup of lanthipeptide RiPPs with antibacterial activity that includes cinnamycin B (30), duramycin (31), duramycin B and C (32), and mathermycin (33) (Fig. 1A). These molecules are produced by actinomycetes and comprise 19 amino acid residues, several of which are modified to generate lanthionine or methyllanthionine cross-links (28, 29). Additional modifications include β-hydroxylation of the invariant aspartic acid residue at position 15 and formation of an unusual lysinoalanine cross-link between the serine residue at position 6 and lysine residue at position 19 (34–36). The interaction of these molecules with PE has therapeutic potential: duramycin binds to human lung epithelial cell membranes leading to changes in the membrane or its components, promoting chloride ion secretion and clearance of mucus from the lungs (27). On this basis, duramycin entered phase II clinical trials for the treatment of cystic fibrosis (37).

Here, we describe activation of the cryptic *Saccharopolyspora* lanthipeptide BGCs and the characterization of their product, a new class II lantibiotic that we called kyamicin. We also exemplify a heterologous expression platform for lanthipeptide production that may be particularly useful for strains that are refractory to genetic manipulation. The methodologies reported should be applicable for the activation of cryptic BGCs from a wide range of actinomycetes.

(This article was submitted to an online preprint archive [38].)

## RESULTS

### Origin, characteristics, and genome sequencing of *Saccharopolyspora* strains.

The *Saccharopolyspora* strains were isolated from ants taken from the domatia of *T. penzigi* plant ants collected in two locations in Kenya (13) and named KY3, KY7, and KY21. The 16S rRNA gene was amplified and Sanger sequenced using universal primers (GenBank accession numbers JX306001, JX306003, and JX306004, respectively). Alignments show that KY3 and KY7 are identical across the sequenced 16S rRNA gene region, while KY21 differs by a single base pair (see Fig. S1 in the supplemental material). Further analysis showed that all three strains share 99% sequence identity with *Saccharopolyspora* 16S rRNA gene sequences in public databases. High-molecular-weight genomic DNA was isolated from each strain, sequenced at the Earlham Institute (Norwich, UK) using SMRT sequencing technology (Pacific Biosciences RSII platform), and assembled using the HGAP2 pipeline as described previously (39). This gave three circular chromosomes of approximately 6.33 Mbp, the full analysis of which will be reported separately. Alignment of the KY3 and KY7 genome sequences using RAST SEED Viewer and BLAST dot plot revealed a full synteny along their genomes with 99% to 100% sequence identity at the nucleotide level, suggesting KY3 and KY7 are the same strain and differ from KY21.

### Identification of a conserved cinnamycin-like BGC.

The biosynthetic potential of all three strains was probed using the genome mining platform antiSMASH (40). The three genomes each harbor approximately 25 BGCs with significant overlap. Among these was a BGC for a cinnamycin-like lanthipeptide. The BGCs from strains KY3 and KY7 were identical and share 98% identity with that from KY21. They have an identical propeptide sequence encoded by the precursor peptide gene, suggesting they all encode the same molecule which we named kyamicin (Fig. 1B). The sequence and annotations for these three BGCs have been deposited at GenBank under the accession numbers MK251551 (KY3) and MK251553 (KY21).

Through comparison to the cinnamycin BGC (26) and cinnamycin biosynthesis (34), we assigned roles to each of the genes in the kyamicin (kya) BGC (Table 1). The kya BGC is more compact than the cinnamycin one, and the genes missing from the kyamicin BGC are dispensable for cinnamycin production (41). The *cinorf11* gene is not required for cinnamycin production, but a homologue is present in the kyamicin cluster. While *cinorf11* lacks a plausible stop codon and its reading frame extends 570 bp into the *cinR1* gene, its homologue, *kyaorf11*, has a stop codon and does not run into the *kyaR1* gene, suggesting it may encode a functional protein.

**TABLE 1:** Proteins encoded by the kyamicin, cinnamycin, and duramycin BGCs

Kyamicin BGC (no. of aa)	Cinnamycin BGC (no. of aa)	Duramycin BGC (no. of aa)	Proposed function
KyaN (123)	CinN (119)	DurN (119)	Formation of lysinoalanine bridge
KyaA (78)	CinA (78)	DurA (77)	Precursor peptide
KyaM (1,065)	CinM (1,088)	DurM (1,083)	Formation of lanthionine residues
KyaX (302)	CinX (325)	DurX (327)	Hydroxylation of Asp15
KyaT (327)	CinT (309)	DurT (352)	Export
KyaH (294)	CinH (290)	DurH (290)	Export
Not present	CinY	DurY	Not essential
Not present	CinZ	DurZ	Not essential
Not present	Cinorf8	Durorf8	Not essential
Not present	Cinorf9	Not present	Not essential
KyaR (216)	CinR (216)	DurR (216)	Regulation
KyaK (372)	CinK (354)	DurK (349)	Regulation
KyaL (226)	CinL (236)	DurL (235)	Immunity
Kyaorf11 (295)	Cinorf11 (396)	Durorf11 (396)	Not essential
KyaR1 (260)	CinR1 (261)	DurR1 (261)	Regulation

To detect production of kyamicin, we grew all three strains on a range of 13 liquid media (see Table S1) and collected extracts after 4, 5, 6, and 7 days of growth by using (individually) methanol and ethyl acetate. Analysis of the extracts using ultraperformance liquid chromatography-mass spectrometry (UPLC-MS) failed to identify the anticipated product (the methods were validated using authentic duramycin). This was consistent with parallel bioassays which failed to show any antibacterial activity for the extracts against Bacillus subtilis EC1524, which is sensitive to cinnamycin (26). Similarly, no activity was observed in overlay bioassays.

### Activation of the kyamicin BGC.

**FIG 2:**
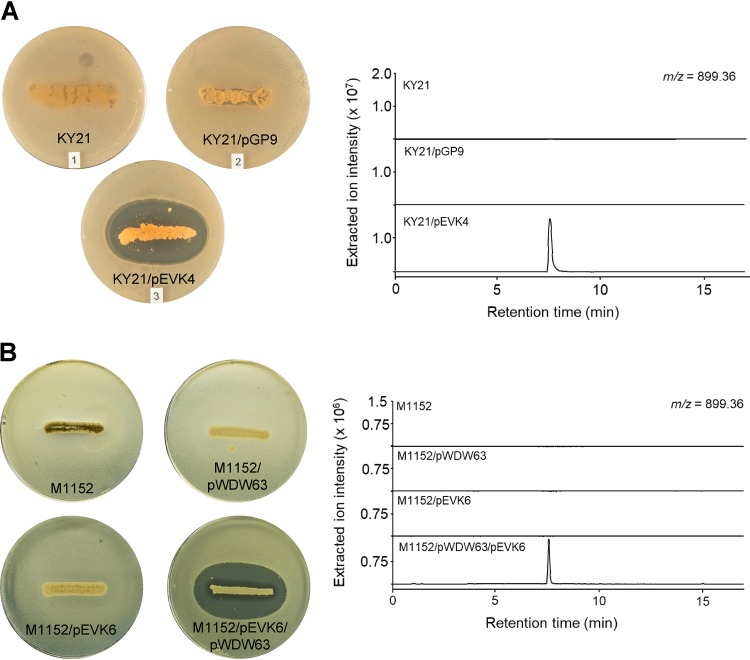
Activation of kyamicin biosynthesis and heterologous expression. Overlay bioassays were carried out with B. subtilis EC1524, and agar plugs were taken adjacent to the central streak and analyzed by UPLC-MS. Extracted ion chromatograms are shown for *m/z* 899.36 ([M + 2H]^2+^). Images and LC traces are representative of at least three biological repeats. (A) Activation of kyamicin production in KY21 strains. The pEVK4 vector containing *kyaR1* and *kyaL* results in a zone of inhibition, corresponding to the production of kyamicin, in contrast to the pGP9 empty vector control or the wild-type strain. (B) Heterologous expression of kyamicin in S. coelicolor M1152. A zone of inhibition, corresponding to kyamicin production, is observed only when pWDW63 carrying the kya biosynthetic genes is expressed in combination with pEVK6 carrying *kyaR1* and *kyaL*.

Cinnamycin production and immunity ultimately rely on two gene products (41). The transcription of the biosynthetic genes is driven by CinR1, one of the *Streptomyces* antibiotic regulatory proteins (SARP) (which usually act as pathway-specific transcription activators), and immunity is conferred by a methyltransferase (CinL) that modifies PE in the membrane to prevent binding of cinnamycin. We reasoned that transcription of the homologues of these two genes (*kyaR1* and *kyaL*, respectively), driven by a constitutive promoter, would circumvent the natural regulatory mechanism and initiate production of kyamicin. To achieve this, we made a synthetic construct, pEVK1, containing *kyaR1-kyaL* (in that order) (see Fig. S2A). The *kyaR1-kyaL* cassette was cloned into pGP9 (42) to yield pEVK4, which was introduced into the three *Saccharopolyspora* strains by conjugation. This resulted in single copies of the plasmid integrated at the φBT1 phage integration site of each strain. Exconjugants were assayed by overlaying with B. subtilis EC1524, revealing zones of clearing for all three strains containing pEVK4 (Fig. 2A; see also Fig. S3). For the KY21 exconjugant, agar plugs were taken from the zone of clearing, extracted with 5% formic acid, and analyzed by UPLC-MS (Fig. 2A). In contrast to the relevant controls, an ion at *m/z* 899.36 was observed corresponding to the expected [M + 2H]^2+^ ion of kyamicin (Table 2).

**TABLE 2:** Calculated and observed *m/z* values for lantibiotic compounds in this study

Compound	Formula	[M + 2H]^2+^ *m/z*		Error (ppm)
		Calculated	Observed	
Kyamicin	C_76_H_108_N_20_O_25_S_3_	899.3551	899.3553	0.22
Deoxykyamicin	C_76_H_108_N_20_O_24_S_3_	891.3576	891.3557	−2.13
Partially reduced kyamicin	C_76_H_110_N_20_O_25_S_2_	884.3768	884.3767	−0.11
Partially reduced kyamicin	C_76_H_112_N_20_O_25_S	869.3987	869.3990	0.35
Reduced kyamicin	C_76_H_114_N_20_O_25_	854.4204	854.4202	−0.23
Duramycin	C_89_H_125_N_23_O_25_S_3_	1,006.9262	1,006.9232	−2.98
Deoxyduramycin	C_89_H_125_N_23_O_24_S_3_	998.9287	998.9253	−3.40

### Heterologous expression of the kyamicin BGC.

Attempts to scale up cultures of *Saccharopolyspora* sp. KY21/pEVK4 to generate sufficient material for further study were not successful due to low titers and poor growth of the strain. Consequently, we attempted heterologous expression of the kya BGC in the well-established host Streptomyces coelicolor M1152 (43). To achieve this, we cloned *kyaR1L* as an NdeI/HindIII fragment into pIJ10257 (44); this yielded pEVK6, which has the constitutive ermE* promoter driving expression of *kyaR1L*. We then commissioned a synthetic operon containing the regions harboring *kyaN* to *kyaH* plus the upstream promoter region of *kyaN* as an EcoRI/XbaI fragment (Fig. S2B). This was cloned into pSET152 (45) to give pWDW63, which integrates into the S. coelicolor chromosome at the φC31 integration site, conferring apramycin resistance. pEVK6 and pWDW63 were then introduced sequentially into S. coelicolor M1152 via conjugation, and apramycin- plus hygromycin-resistant exconjugants were grown on R5 agar and overlaid with B. subtilis EC1524. In contrast to the control strains, these gave a pronounced zone of clearing. Agar plugs were taken from the zone of clearing, extracted, and analyzed by UPLC-MS, revealing the expected [M + 2H]^2+^ ion for kyamicin which was not present in the controls (Fig. 2B). In addition to kyamicin, a second minor new compound was observed with an *m/z* value of 891.36, consistent with the production of a small amount of deoxykyamicin, presumably reflecting incomplete β-hydroxylation of the aspartic acid residue at position 15 (Table 2 and Fig. S4).

Having established the production of kyamicin in the M1152 heterologous host, we used this system to better understand how each gene product contributes to the activation of kyamicin biosynthesis. We cloned *kyaL* and *kyaR1* separately into pIJ10257 to give pEVK12 and pEVK13, respectively. Each plasmid was then introduced into M1152 alongside pWDW63, and doubly antibiotic-resistant exconjugants were selected. These were grown on R5 agar plates and overlaid with B. subtilis EC1524; agar plugs were extracted from the resulting bioassay plates as before. For M1152/pEVK12 (*kyaL* only) no growth inhibition of the bioassay strain was observed and we could not detect kyamicin or deoxykyamicin using UPLC-MS. For M1152/pEVK13 (*kyaR1* only), we observed a zone of inhibition which was approximately three times smaller than for the M1152/pEVK6 (*kyaR1L*) positive control. UPLC-MS analysis of the M1152/pEVK13 strain detected only deoxykyamicin (Fig. S4). No significant change in overall titers was observed between these strains, and we ruled out the possibility of suppresser mutations in *kyaX* (encoding the hydroxylase) by PCR amplification and sequencing of the DNA encoding this gene and the surrounding region (data not shown). This is consistent with previous work which reported that deoxy versions of lantibiotics have lower biological activity (46).

### Isolation, structure elucidation, and bioactivity.

To isolate and verify the structure of kyamicin, growth of S. coelicolor M1152/pEVK6/pWDW63 was scaled up in liquid culture, and the cell pellet was extracted with 50% methanol. Crude extracts were further purified using semipreparative high-performance liquid chromatography (HPLC) to yield pure kyamicin (2.5 mg).

**FIG 3:**
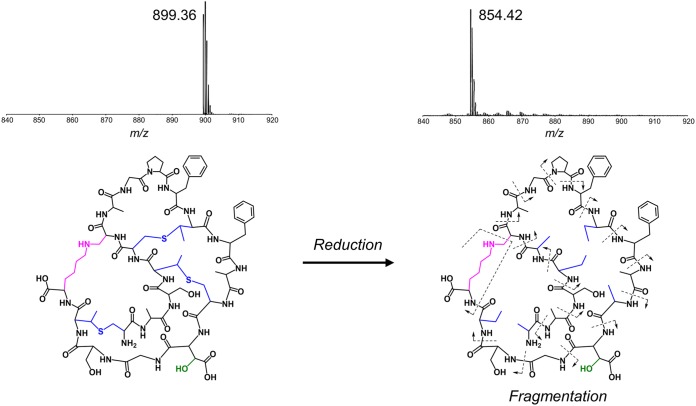
Characterization of kyamicin. The connectivity of the peptide was confirmed by chemical reduction followed by tandem MS fragmentation. Reduction with NaBH_4_-NiCl_2_ resulted in the cleavage of the methyllanthionine bridges (blue), corresponding to the loss of three S atoms and gain of six H atoms, with a mass shift from *m/z* 899.36 ([M + 2H]^2+^) to *m/z* 854.42 ([M + 2H]^2+^). Tandem MS using the MALDI-TOF LIFT method allowed identification of the *y* ion (NH_3_^+^) series for the complete peptide (see Fig. S5 in the supplemental material). Fragmentation of the lysinoalanine bridge (pink) occurred via rearrangement to give N=CH_2_ at the terminus of the lysine side chain and a glycine residue at position 6.

As the methyllanthionine bridges of kyamicin limit the ability to induce fragmentation in tandem mass spectrometry (MS/MS) experiments, the lantibiotic was subjected to chemical reduction with NaBH_4_-NiCl_2_ using a procedure published previously for the related molecule cinnamycin B (30). This leads to removal of the methyllanthionine bridges, and as anticipated, UPLC-MS of the product molecule showed an [M + 2H]^2+^ ion at *m/z* 854.42 corresponding to the loss of three sulfur atoms and gain of six hydrogen atoms (Table 2 and Fig. 3). Tandem MS experiments were carried out using both electrospray ionization (ESI) and matrix-assisted laser desorption ionization–time of flight (MALDI-TOF) methods. While ESI gave a complex mixture of fragmentation ions, for MALDI-TOF, the *y* ion (NH_3_^+^) series could be clearly observed, with fragmentation at the lysinoalanine bridge appearing to occur via a rearrangement to give a glycine residue at position 6 and N=CH_2_ at the end of the lysine side chain (see Fig. S5). The connectivity of the peptide was consistent with the primary sequence of kyamicin predicted by our bioinformatics analysis.

The chemical structure was further examined by nuclear magnetic resonance (NMR) experiments comprising ^1^H, heteronuclear single quantum coherence (HSQC) spectroscopy, total correlation spectroscopy (TOCSY), and nuclear Overhauser effect spectroscopy (NOESY) analyses. Overall, 14 spin systems were partially or completely identified in the TOCSY spectrum. These were putatively assigned based on their spatial relationship determined from the NOESY spectrum. Coupling in the HSQC spectrum then allowed identification of several C atoms in the molecule. Spectra and assignments can be found in Fig. S6 and Table S2.

**FIG 4:**
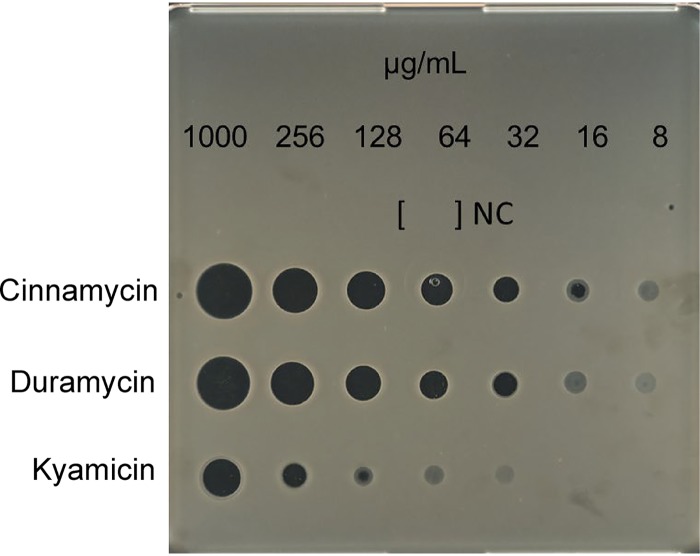
Comparative bioassay of kyamicin, duramycin, and cinnamycin against B. subtilis EC1524. The MIC of each substance was determined by direct application of serial dilutions of the compounds in water on a SNA plate inoculated with B. subtilis EC1524. NC, H_2_O (negative control). Kyamicin displays an MIC of 128 μg/ml, whereas duramycin inhibits at 32 μg/ml and cinnamycin at 16 μg/ml.

The bioactivity of the purified compound was compared with cinnamycin and duramycin using the spot-on-lawn method. The MIC of kyamicin against B. subtilis EC1524 was 128 μg/ml, whereas duramycin inhibited at 32 μg/ml and cinnamycin at 16 μg/ml, representing 4- and 8-fold MIC increases, respectively (Fig. 4).

### Cross-species activation of the duramycin BGC.

Many cinnamycin-like BGCs can be identified in the published sequence databases, but their products remain cryptic. Thus, the potential of the *kyaR1-kyaL* construct to induce expression of other cinnamycin-like lantibiotics was explored.

**FIG 5:**
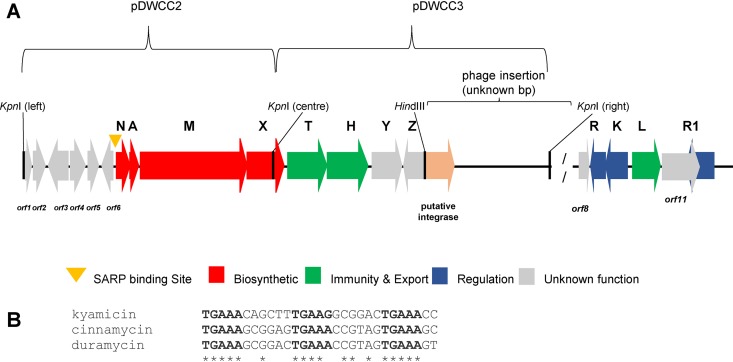
Schematic of duramycin BGC and plasmids used to construct pOJKKH, and SARP binding sites of kyamicin, cinnamycin, and duramycin. (A) The *S. cinnamoneus* DNA sequences represented on the plasmids pDWCC2 and pDWCC3 are present in the published genome sequence as bp 81593 to 99144 of contig NZ_MOEP01000024.1. pDWCC2 consists of the area from the left KpnI site (from *durorf1*) to the central KpnI site in *durX*. pDWCC3 consists of the area covering from the central KpnI site in *durX* to the right-side KpnI site after a putative integrase-encoding gene. The putative duramycin resistance/regulatory genes are represented in the published genome sequence by bp 54637 to 59121 of contig NZ_MOEP01000113.1. (B) Sequence alignment of putative SARP binding sites of kyamicin, cinnamycin, and duramycin. Conserved residues within all three sequences are marked with asterisks, and the 5-bp SARP binding motifs are in bold font. The alignment was performed with Clustal Omega (v1.2.4).

**FIG 6:**
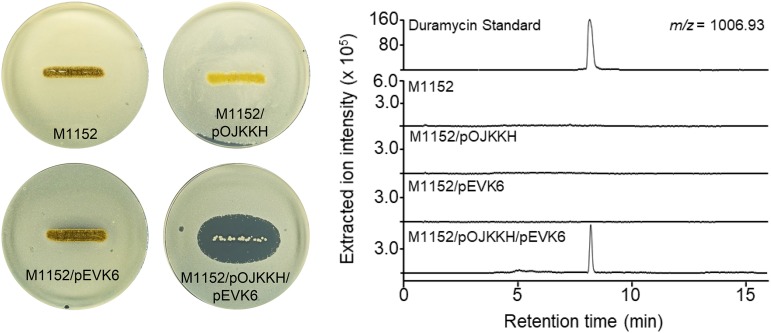
Activation of duramycin biosynthesis. Overlay bioassays were carried out with B. subtilis EC1524, and agar plugs were taken adjacent to the central streak and analyzed by UPLC-MS. Extracted ion chromatograms are shown for *m/z *1,006.93 ([M + 2H]^2+^). Duramycin was only detected in the strain carrying both pOJKKH and pEVK6. The duramycin peak aligns with an authentic standard of duramycin (0.1 mg/ml in 5% formic acid), shown on a separate scale. Images and LC traces are representative of at least three biological repeats.

The BGC for duramycin was cloned previously from Streptomyces cinnamoneus ATCC 12686 (Fig. 5A), but attempts to produce the lantibiotic heterologously failed. Consequently, the duramycin BGC was reconfigured in pOJKKH, which contains all the biosynthetic genes but lacks immunity and regulatory genes and has a SARP binding site upstream of *durN* that is similar to that upstream of *kyaN* (Fig. 5B) (41). pOJKKH and pEVK6 were introduced sequentially into S. coelicolor M1152 via conjugation and the resulting exconjugants assessed for duramycin production. Overlay bioassays using B. subtilis EC1524 indicated the production of an antibacterial molecule by S. coelicolor M1152/pOJKKH/pEVK6 (Fig. 6). Agar within the growth inhibition zone was extracted and the resulting sample analyzed by UPLC-MS. An ion at *m/z* 1,006.92 was observed, corresponding to the expected [M + 2H]^2+^ ion for duramycin (Table 2). The production of duramycin was confirmed by comparison to an authentic standard. A deoxy derivative was also detected with an *m/z* of 998.93 (Table 2), typically at ∼30% the level of duramycin. Expression of pOJKKH alone or in conjunction with the empty pIJ10257 vector did not result in duramycin biosynthesis, demonstrating that expression of both *kyaR1* and *kyaL* is required to induce heterologous duramycin biosynthesis in S. coelicolor M1152. Thus, we have shown that the SARP and immunity genes from a cinnamycin-like BGC from a *Saccharopolyspora* species can be used to activate a cinnamycin-like BGC from a *Streptomyces* species, a cross-genus activation.

## DISCUSSION

Three isolates from the rare actinomycete genus *Saccharopolyspora* were isolated from the external microbiome of *T. penzigi* plant ants collected at two locations in Kenya more than 50 km apart (13). Despite this geographical separation, their genomes were extremely similar, and analysis using antiSMASH identified almost identical biosynthetic capabilities. Among the conserved BGCs was one encoding a cinnamycin-like lantibiotic, which we named kyamicin.

Despite culturing on a wide range of media, we were unable to elicit production of kyamicin in the wild-type *Saccharopolyspora* strains. The production of cinnamycin in *S. cinnamoneus* DSM 40005 requires the expression of two key genes, *cinR1* and *cinL*, encoding a pathway-specific regulatory protein (a SARP) and an immunity protein (a PE methyltransferase), respectively (41). As the *kya* BGC contains homologues of these genes, we expressed them constitutively in the three *Saccharopolyspora* strains, which led to activation of the BGC and production of kyamicin. Since we were unable to isolate enough kyamicin from these strains for further study, a heterologous production platform was developed using S. coelicolor M1152, which allowed us to confirm the structure of kyamicin and assess its antibacterial activity. The chemical structure of kyamicin differs from that of cinnamycin and duramycin at 6 of the 19 amino acid residues but not at any involved in formation of the lanthionine or lysinoalanine bridges.

Having demonstrated the utility of a constitutively expressed SARP/immunity cassette for driving expression of the otherwise silent *kya* BGC, we utilized this knowledge to activate duramycin production in a heterologous host. Contemporaneous with our experiments, the duramycin BGC was also identified by genome sequencing of *S. cinnamoneus* ATCC 12686 (35). This analysis described the same genomic region containing *durN* to *durH* and surrounding genes (Table 1) but failed to reveal putative regulatory and immunity genes. Coexpression of *durA*, *durM*, *durN*, and *durX* in Escherichia coli was sufficient to direct the biosynthesis of duramycin A, and the functions of DurA, DurM, DurN, and DurX were confirmed by detailed biochemical analyses. Our subsequent bioinformatic analysis of the published genome sequence identified homologs of the immunity genes *cinL* and *kyaL* and the regulatory genes *cinRKR1* and *kyaRKR1* in the region from bp 54637 to 59121 of contig MOEP01000113.1 from the deposited genome sequence (accession no. NZ_MOEP00000000). This region is separated from the *dur* biosynthetic genes by a section of DNA with low GC content, the analysis of which suggests that a phage or other mobile element may have inserted between *durZ* and *durorf8* (Fig. 5). Thus, it appears likely that the immunity and regulatory mechanisms described previously for the control of cinnamycin biosynthesis are conserved for duramycin biosynthesis in *S. cinnamoneus* ATCC 12686.

Given the potential utility of cinnamycin-like class II lanthipeptides in several therapeutic contexts, the ability to generate analogues of these compounds with modified properties and in sufficient quantity for preclinical assessment is of significant value. The methods described here provide a platform for the identification of additional natural lanthipeptides whose biosynthesis cannot be detected in the host strain and for the diversification of their chemical structures to generate new-to-nature molecules.

## MATERIALS AND METHODS

### Bacterial strains, plasmids, and growth conditions.

All bacterial strains and plasmids used in this study are listed in Table 3. *Saccharopolyspora* and *Streptomyces* strains were grown on soya flour-mannitol (SFM) agar medium with appropriate antibiotics at 30°C unless otherwise stated. E. coli and B. subtilis EC1524 strains were grown on lysogeny broth (LB) medium with appropriate antibiotics at 37°C. R5 agar (47) was used for bioassay plates.

**TABLE 3:** Strains and plasmids used in this work

Strain or plasmid	Description	Reference or source
Strains		
*Saccharopolyspora* sp.		
KY3	Strain from the cuticles of *Tetraponera penzigi*	This work
KY3/pGP9	KY3 strain carrying the empty pGP9 plasmid	This work
KY3/pEVK4	KY3 strain carrying pEVK4, which activates kyamicin production	This work
KY7	Strain from the cuticles of *Tetraponera penzigi*	This work
KY7/pGP9	KY7 strain carrying the empty pGP9 plasmid	This work
KY7/pEVK4	KY7 strain carrying pEVK4, which activates kyamicin production	This work
KY21	Strain from the cuticles of *Tetraponera penzigi*	This work
KY21/pGP9	KY21 strain carrying the empty pGP9 plasmid	This work
KY21/pEVK4	KY21 strain carrying pEVK4, which activates kyamicin production	This work
Streptomyces coelicolor	Non-antibiotic-producing superhost, Δ*act* Δ*red* Δ*cpk* Δ*cda rpoB*(C1298T)	43
M1152/pEVK6	M1152 carrying pEVK6	This work
M1152/pWDW63	M1152 carrying the kyamicin biosynthetic genes	This work
M1152/pEVK6/pWDW63	M1152 carrying the kyamicin biosynthetic genes and pEVK6, which activates kyamicin production	This work
M1152/pWDW63/pEVK12	M1152 carrying the kyamicin biosynthetic genes pEVK12 for constitutive expression of *kyaL*	This work
M1152/pWDW63/pEVK13	M1152 carrying the kyamicin biosynthetic genes pEVK13 for constitutive expression of *kyaR1*	This work
M1152/pOJKKH	M1152 carrying the duramycin biosynthetic genes	This work
M1152/pEVK6/pOJKKH	M1152 carrying the duramycin biosynthetic genes and pEVK6, which activates duramycin production	This work
Bacillus subtilis EC1524	Bioassay strain; trpC2, subtilin BGC deleted	26
Plasmids		
pGP9	pSET152-derived φBT-based integrative expression vector	42
pIJ10257	oriT, φBT1 attB-int, Hygr, ermEp*	44
pSET152	ϕC31 attP-conjugative vector	45
pEVK1	pUC57/R1L Synthetic construct with *kyaR1L* genes	GenScript
pEVK4	pGP9/R1L for constitutive expression of *kyaR1L* in *Saccharopolyspora*	This work
pEVK6	pIJ10257/R1L for constitutive expression of *kyaR1L* in S. coelicolor	This work
pEVK12	pIJ10257/L for constitutive expression of *kyaL* in S. coelicolor	This work
pEVK13	pIJ10257/R1 for constitutive expression of *kyaR1* in S. coelicolor	This work
pWDW60	pUC57/Kya synthetic construct with the kyamicin biosynthetic genes *kyaN* to *kyaH*	GenScript
pWDW63	pSET152/Kya for constitutive expression of the kyamicin biosynthetic genes in S. coelicolor	This work
pOJKKH	pOJ436-based plasmid carrying the duramycin biosynthetic genes *durN* to *durZ*	This work

### DNA extraction and genomic analysis.

The salting-out method (47) was used to extract genomic DNA. The DNA was sequenced at the Earlham Institute (Norwich, UK) using SMRT sequencing technology (Pacific Biosciences RSII platform) and assembled using the HGAP2 pipeline (39).

### Overlay bioassays.

For each strain to be tested, a streak from a spore stock was applied in the center of an R5 agar plate and left to grow for 7 days. B. subtilis EC1524 was grown from a single colony overnight and then diluted 1:20 in fresh medium and grown until an optical density at 600 nm (OD_600_) of 0.4 to 0.6. The exponential culture was mixed with 1:100 molten soft nutrient agar (SNA) (47), and the mixture was used to overlay the plate (5 ml SNA mixture/agar plate). The plate was incubated at room temperature overnight.

### Extractions from overlay bioassays.

Plugs of agar 6.35 mm in diameter were taken adjacent to the streaked actinomycete strain on an overlay bioassay plate, corresponding to the zone of growth inhibition where one was observed. Agar plugs were frozen at −80°C for 10 min and thawed, and then 300 μl of 5% formic acid was added. This was vortexed briefly and shaken for 20 min. After centrifugation (15,682 × *g* for 15 min), the supernatant was collected and filtered using a filter vial (HSTL Labs) prior to UPLC-MS analysis.

### UPLC-MS.

Data were acquired with an Acquity UPLC system (Waters) equipped with an Acquity UPLC BEH C_18_ column, 1.7 μm, 1 mm by 100 mm (Waters), connected to a Synapt G2-Si high-resolution mass spectrometer (Waters). For analytical UPLC, 5.0 μl of each sample was injected and eluted with mobile phases A (water-0.1% formic acid) and B (acetonitrile-0.1% formic acid) at a flow rate of 80 μl/min. Initial conditions were 1% B for 1.0 min, ramped to 40% B within 9.0 min, ramped to 99% B within 1.0 min, held for 2 min, returned to 1% B within 0.1 min, and held for 4.9 min.

MS spectra were acquired with a scan time of 1.0 s in the range of *m/z *50 to 2,000 in positive-resolution mode. The following parameters were used: capillary voltage, 3.0 kV; cone voltage, 40 V; source offset, 80 V; source temperature, 130°C; desolvation temperature, 350°C; desolvation gas flow, 700 liters/h. A solution of sodium formate was used for calibration. Leucine encephalin peptide (H_2_O-methanol [MeOH]-formic acid [49.95:49.95:0.1]) was used as lock mass (556.2766 *m/z*) and was injected every 30 s during each run. The lock mass correction was applied during data analysis.

### Design of kya BGC activation and immunity plasmids.

pEVK1, a pUC57 derivative, contains the synthetic *kyaR1* and *kyaL* (GenScript) arranged as an operon. pEVK1 has an NdeI site overlapping the start codon of *kyaR1* and a HindIII site immediately after the stop codon of *kyaL*, with the two genes separated by a short intergenic region containing a ribosome binding site (RBS) designed from the RBS of *cinN* (chosen because its sequence is most similar in the BGC to that of an ideal RBS) (see Fig. S2A in the supplemental material). The NdeI-HindIII *kyaR1L* fragment from pEVK1 was cloned into pGP9 (42) to give pEVK4, and into pIJ10257, a φBT1-based integrative expression vector with a hygromycin resistance marker (44), to give pEVK6. *kyaR1* and *kyaL* were amplified individually as NdeI-HindIII-compatible fragments using the primers AmplkyaR1-F (GCGCAAGCTTCTACGACGCGGTGTGA) and AmplkyaR1-R (GCGCGCCATATGAAACCGCTGTCGTTCC) for *kyaR1*, and AmplkyaL-F (GCGCGCCATATGGATCCAGTACAGACCA) and AmplkyaL-R (GCGCAAGCTTTCAGCGGTCCTCCGCC) for *kyaL*; they were cloned as NdeI-HindIII fragments into pIJ10257 to yield pEVK12 and pEVK13, respectively. PCR-generated fragments were verified by Sanger sequencing.

### Cloning the duramycin BGC from Streptomyces cinnamoneus ATCC 12686.

The cloning of an ∼5-kb BglII fragment of chromosomal DNA to create pIJ10100 was described previously (26). This plasmid has a KpnI site in the middle of *durX*. KpnI fragments upstream and downstream of this KpnI site were identified by Southern blotting and isolated by creating a minilibrary of KpnI fragments in pBluescriptIIKS followed by colony hybridization to give pDWCC2 and pDWCC3, respectively. Analysis of the sequence of these plasmids identified 15 genes (shown in Fig. 5). A plasmid carrying the duramycin biosynthetic genes but not the putative phage DNA was prepared by digesting pDWCC3 with XhoI and HindIII (site is in the multiple cloning site of pBluescript II KS) removing the 5′ end of *durZ* and the putative phage DNA. This region was replaced with a XhoI- and HindIII-cut PCR fragment that reconstituted the portion of *dur*Z removed in the previous step and introduced a HindIII site upstream of the *dur*Z start codon. The 666-bp PCR fragment was generated using the primers BK10 (GAGCTTGACGCCGCCGAAGTAGC) and Hindprim (GCGGCGAAGCTTGAGGTGGCCTCCTCCACGAAGCCA) with pDWCC3 as the template and was cut with XhoI plus HindIII to give a 363-bp fragment. The resulting plasmid was then digested with KpnI plus XbaI (the XbaI site is in the multiple cloning site of pBluescript II KS), and the fragment carrying putative duramycin genes was cloned into KpnI-plus-XbaI-cleaved pOJ436 to give pOJKH. The KpnI fragment from pDWCC2 was then cloned into pOJKH cut with KpnI to give pOJKKH, which was verified by BglII digestion, thus restoring the original gene context.

### Isolation and purification of kyamicin.

S. coelicolor M1152/pWDW63/pEVK6 was grown in tryptic soy broth (12 2.5-liter Erlenmeyer flasks, each containing 500 ml) and incubated at 28°C and 200 rpm on an orbital shaker for 7 days. The cells were harvested and extracted with methanol-water (1:1,500 ml) with ultrasonication for 2 h and subsequent shaking for 16 h. After centrifugation, the supernatant was filtered and concentrated under vacuum, giving 613 mg of crude material, which was then purified by semipreparative HPLC. Chromatography was achieved over a Phenomenex Gemini-NX reversed-phase column (C_18_, 110 Å, 150 mm by 21.2 mm) using a Thermo Scientific Dionex UltiMate 3000 HPLC system. A gradient was used with mobile phases A (H_2_O with 0.1% formic acid) and B (methanol): 0 to 1 min, 10% B; 1 to 35 min, 10% to 85% B; 35 to 40 min, 85% to 100% B; 40 to 45 min, 100% B; 45 to 45.1 min, 100% to 10% B; 45.1 to 50 min, 10% B; flow rate, 20 ml/min; injection volume, 1,000 μl. Absorbance was monitored at 215 nm, and fractions (20 ml) were collected and analyzed by UPLC-MS. Kyamicin was observed in fractions 22 to 25, which were combined and concentrated to yield an off-white solid (2.5 mg).

### MIC determination.

The spot-on-lawn method was used to determine lantibiotic MICs. A 1,000-μg/ml stock solution of each lantibiotic was prepared using sterile water, along with serial dilutions from 256 to 8 μg/ml. B. subtilis EC1524 was grown and mixed with molten SNA as described above to create a lawn of bacterial growth. Once set, 5 μl of each dilution was applied directly to the agar and incubated overnight at room temperature. The MIC was defined as the lowest concentration for which a clear zone of inhibition was observed.

### Chemical reduction of kyamicin.

Kyamicin (1 mg) was dissolved in methanol (0.5 ml) and added to an aqueous solution of NiCl_2_ (20 mg/ml; 0.5 ml). The solution was mixed with solid NaBH_4_ (5 mg), resulting in the generation of hydrogen gas and the formation of a black Ni_2_B precipitate. The tube was immediately sealed, and the mixture was stirred at 55°C. The reaction progress was monitored by UPLC-MS as described above, for which a peak with an *m/z* of 899.36 was observed for kyamicin ([M + 2H]^2+^). The successive formation of peaks with the following masses was observed: *m/z *884.38, 869.40, and 854.42, corresponding to the successive reduction of the three thioether bridges. After 5 h, only the ion with *m/z* 854.42 was observed, indicating that the starting material had been completely reduced. The precipitate was collected by centrifugation at 15,682 × *g* for 10 min. As the reaction supernatant contained only trace amounts of the desired product, a fresh solution of MeOH-H_2_O (1:1; 0.5 ml) was added to the precipitate and it was subjected to ultrasonication for 30 min. Reduced kyamicin was then detected in sufficient quantity for MS/MS experiments to confirm the peptide sequence.

### MS analysis of reduced kyamicin.

For ESI-MS^/^MS analysis, the mass of interest (854.42) was selected using an inclusion list and fragmented using data-directed analysis (DDA) with the following parameters: top3 precursor selection (inclusion list only); MS2 threshold, 50,000; scan time, 0.5 s without dynamic exclusion. Collision energy (CE) was ramped between 15 and 20 at low mass (50 *m/z*) and 40 to 100 at high mass (2,000 *m/z*). Further increase of the CE from 20 to 30 and from 60 to 120 led to complete fragmentation.

For MALDI-TOF-MS, the samples were mixed with α-cyano-4-hydroxycinnamic acid as matrix and analyzed on an Autoflex Speed MALDI-TOF/TOF mass spectrometer (Bruker Daltonics GmbH). The instrument was controlled by a flexControl (version 3.4; Bruker) method optimized for peptide detection and calibrated using peptide standards (Bruker). For sequence analysis, fragments produced by post-source decay (PSD) were measured using the LIFT method (Bruker). All spectra were processed in flexAnalysis (version 3.4; Bruker).

### NMR experiments.

NMR measurements were performed on a Bruker Avance III 800-MHz spectrometer. Chemical shifts are reported in parts per million (ppm) relative to the solvent residual peak of DMSO-*d*_6_ (^1^H: 2.50 ppm, quintet; ^13^C: 39.52 ppm, septet).

### Data availability.

The sequence and annotations for these three BGCs have been deposited at GenBank under the accession numbers MK251551 (KY3) and MK251553 (KY21).

## Supplementary Material

Supplemental file 1
