# Supplementary material for: In Situ Activation and Heterologous Production of a Cryptic Lantibiotic from an African Plant Ant-Derived Saccharopolyspora Species
Source: Appl Environ Microbiol. 2020 Jan 21;86(3):e01876-19. doi: 10.1128/AEM.01876-19 (PMC6974636; doi:10.1128/AEM.01876-19)
Supplement: Supplemental file 1 [file zam003209566s1.pdf]

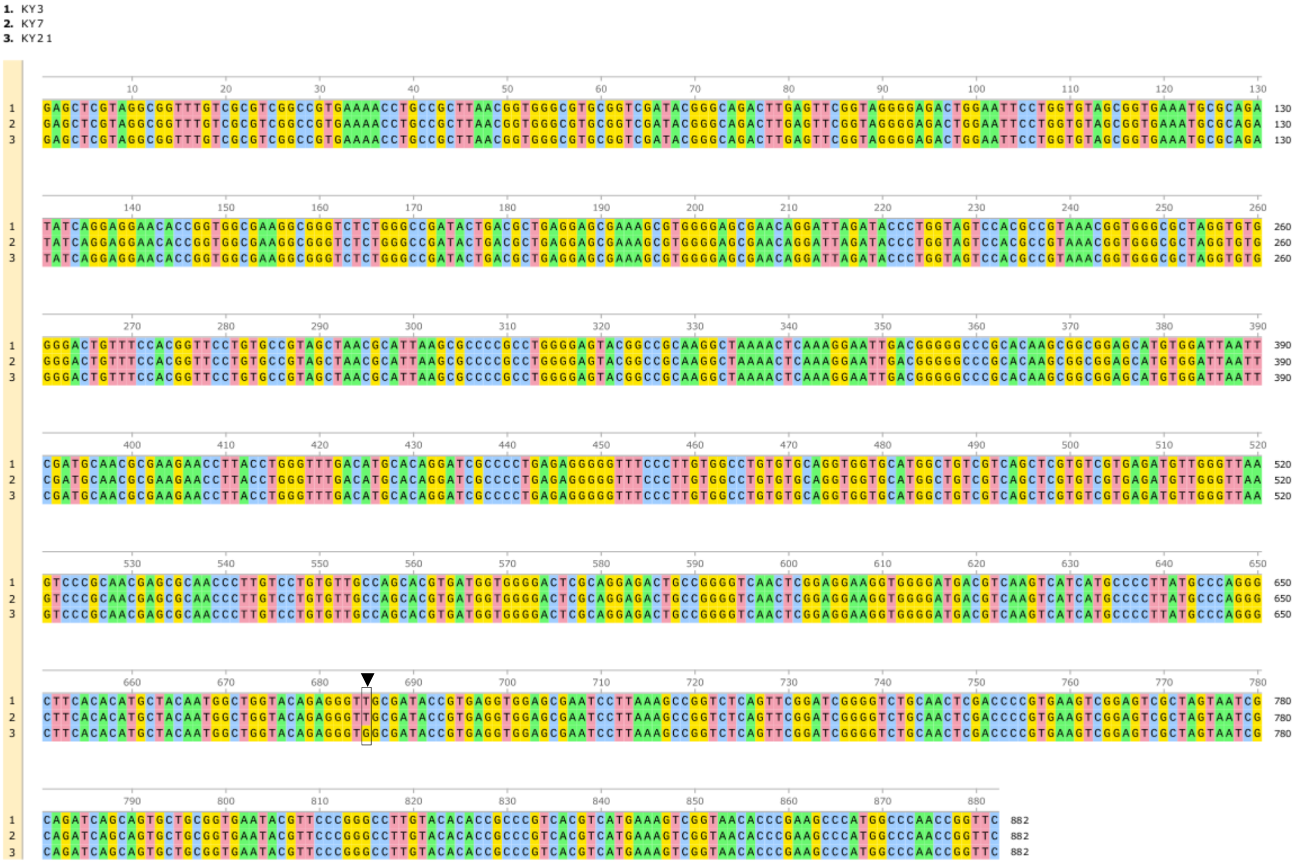

FIG S1 Alignment of *Saccharopolyspora* sp. KY3, KY7 and KY21 16S rDNA sequences. The alignment was performed with Clustal Omega (v1.2.4) and the figure was generated by SnapGene Viewer (v4.2.11). The difference between KY21 to strains KY3 and KY7 is indicated with a black arrow and a box at position 685.

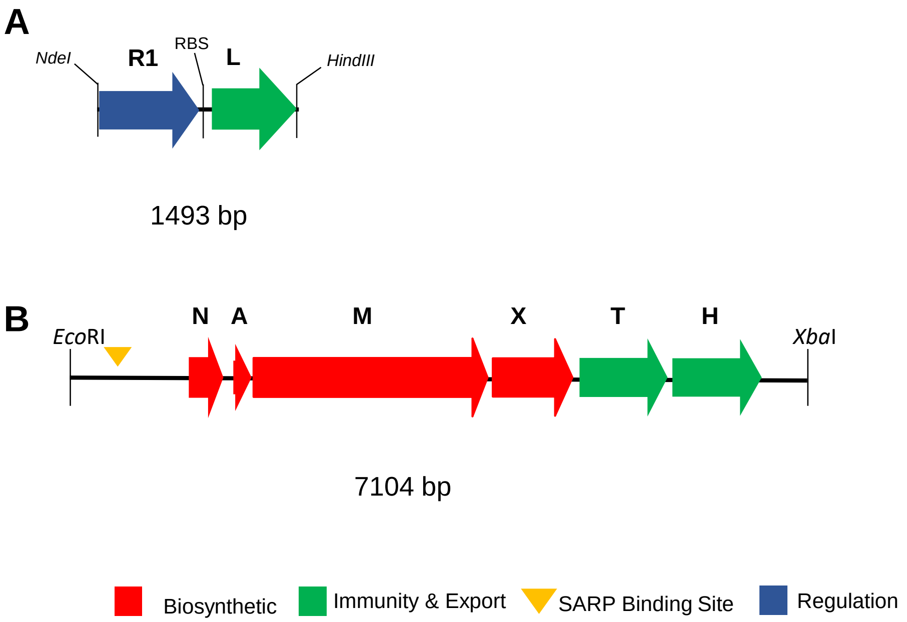

FIG S2 Schematic of synthetic artificial operons. (A) The operon consisting of *kyaR1*, encoding a *Streptomyces* antibiotic regulatory protein (SARP), and *kyaL*, encoding a PE-methyl transferase that provides resistance – the homologues of *cinR1* and *cinorf10* respectively. **(B)** The operon carrying genes *kyaN* to *kyaH* as an *EcoR*I/*Xba*I fragment. These genes are expected to be essential for kyamicin biosynthesis.

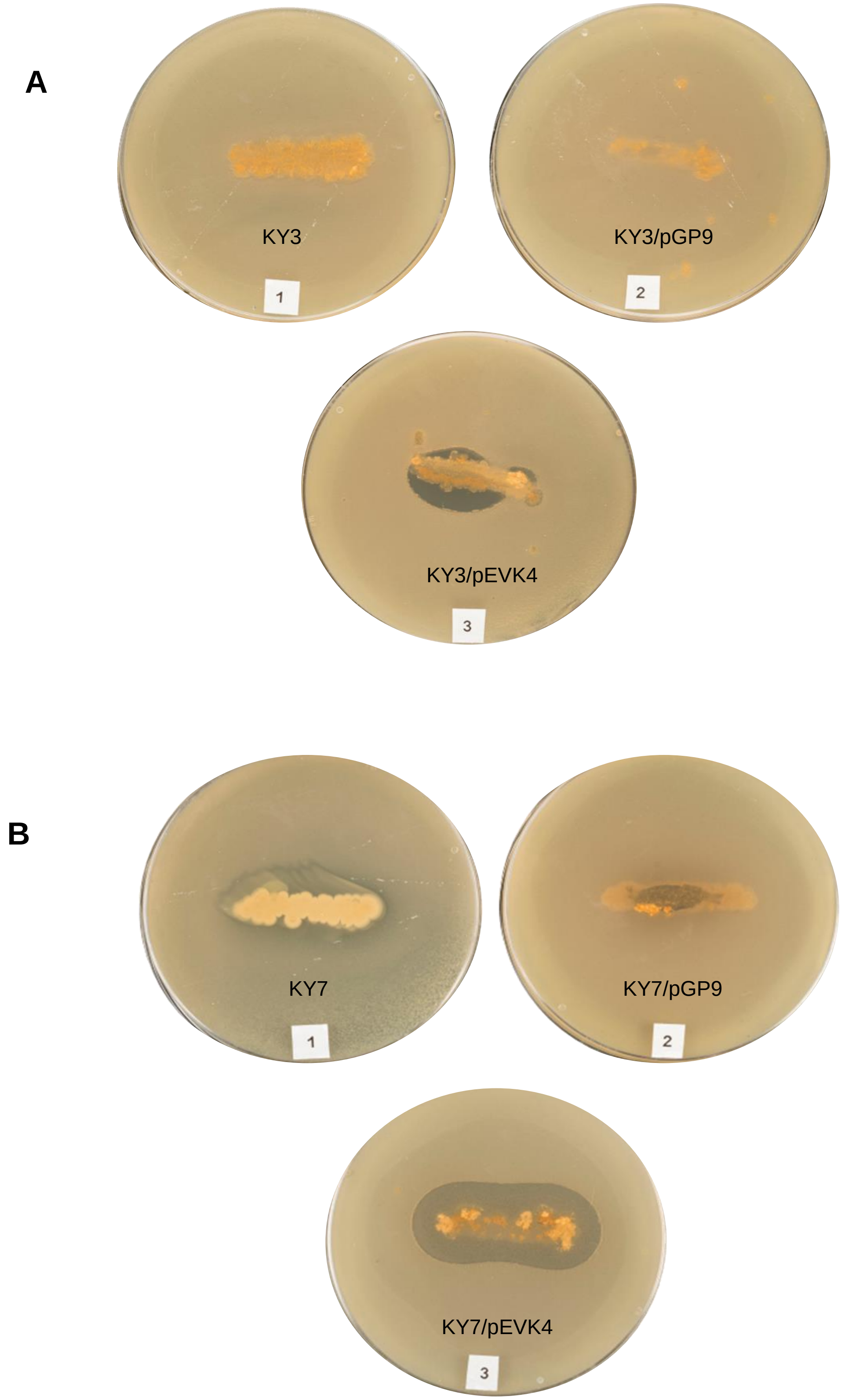

FIG S3 Activation of kyamicin biosynthesis in KY3 and KY7. The pEVK4 vector containing *kyaR1* and *kyaL* results in a zone of inhibition, corresponding to the production of kyamicin, in contrast to the pGP9 empty vector control or the wildtype strain. **(A)** Activation of kyamicin production in KY3, and **(B)** in KY7 strains.

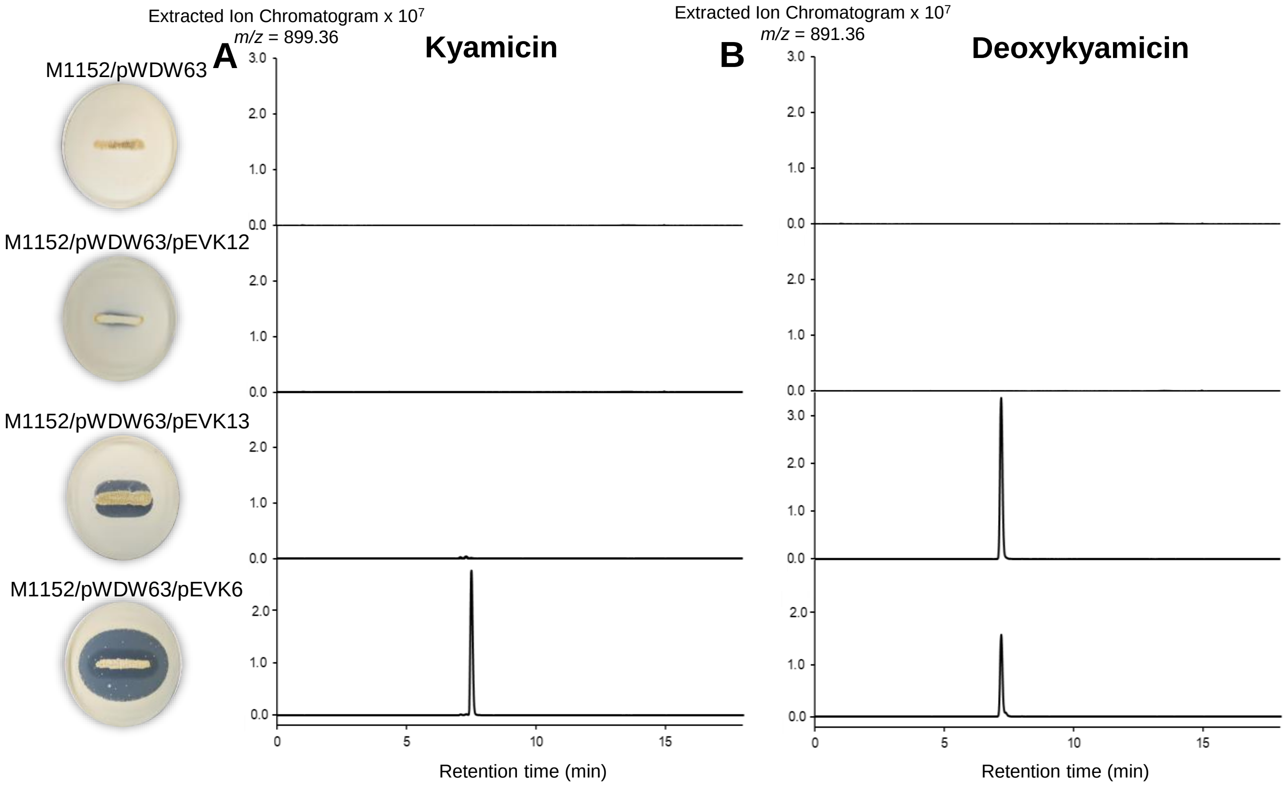

FIG S4 Dissection of the contribution of *kyaR1* and *kyaL* to kyamicin BGC activation. Overlay bioassays were carried out with *B. subtilis* EC1524 and agar plugs were taken adjacent to the central streak and analysed by UPLC/MS. The pEVK12 vector containing only *kyaL* does not result in a zone of inhibition. The pEVK13 vector containing only *kyaR1* results in a zone of inhibition, corresponding to the production of deoxykyamicin only. Expression of both pEVK12 and pEVK13 results in a zone of inhibition, corresponding to the production of both kyamicin and deoxykyamicin. Images and LC traces are representative of at least three biological repeats. **(A)** Extracted ion chromatograms are shown where *m/z* = 899.36 ($[M + 2H]^{2+}$). **(B)** Extracted ion chromatograms are shown where *m/z* = 891.36 ($[M + 2H]^{2+}$).

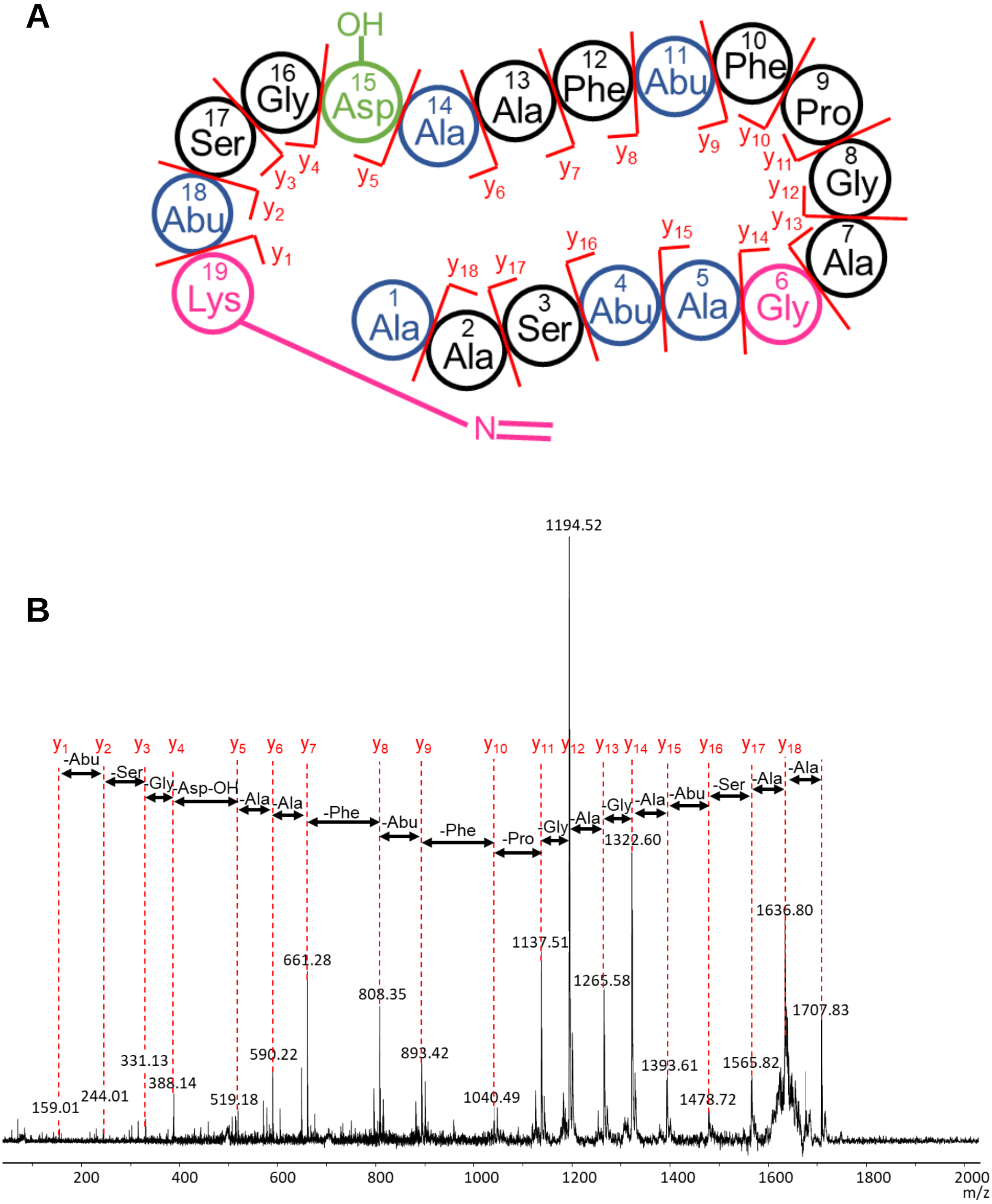

FIG S5 Kyamicin fragmentation. Following reduction to remove methyllanthionine bridges, kyamicin was subject to MALDI-ToF tandem MS, giving the complete *y* ion (NH_3^+) series. **(A)** Structure of reduced kyamicin and the y_1 – y_{18} ion series. **(B)** MALDI-ToF tandem MS spectrum with the *y* ion series indicated with dashed red lines.

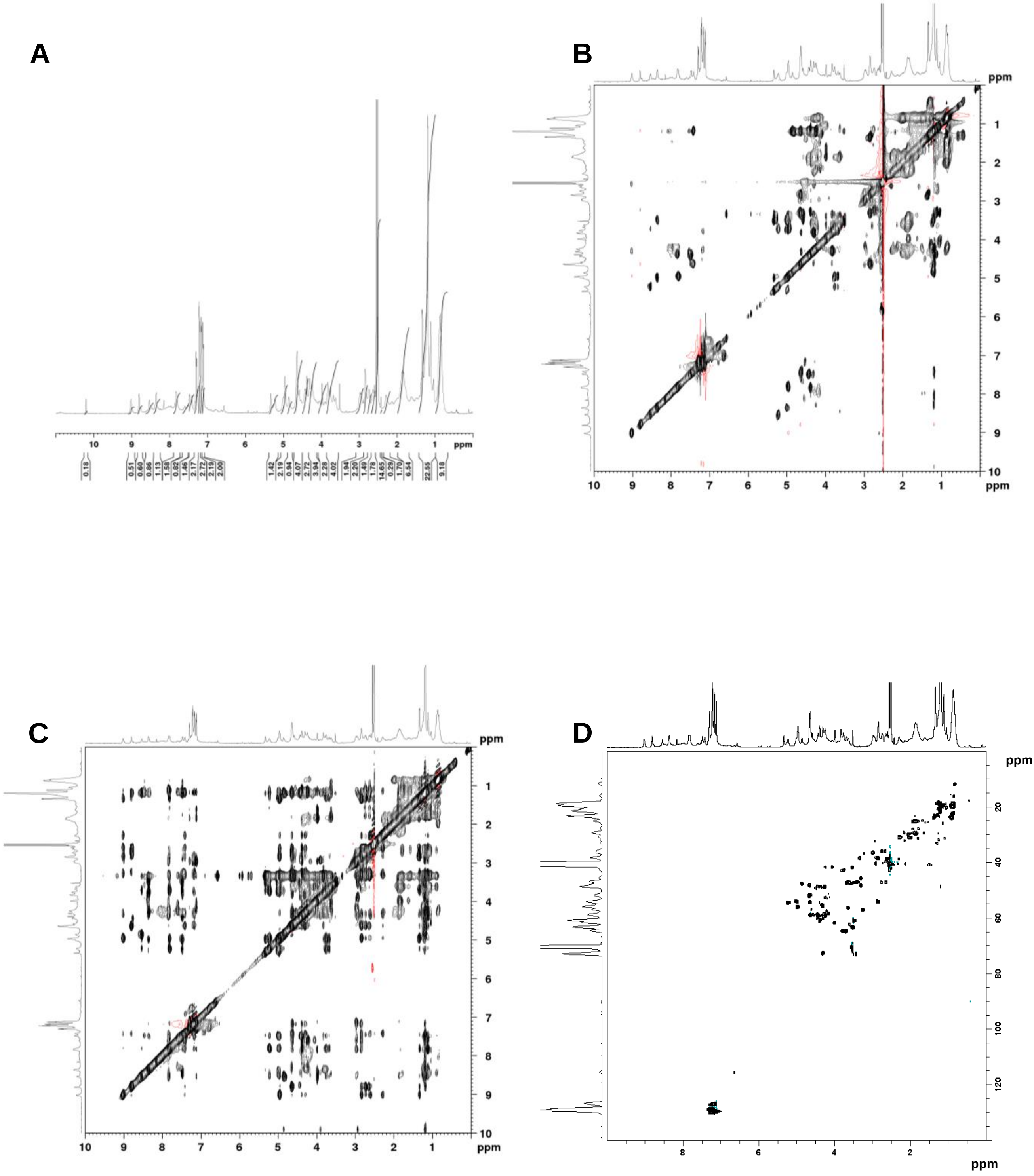

FIG S6 Kyamicin NMR Spectra. (A) ^{1}H NMR spectrum. **(B)** TOCSY spectrum. **(C)** NOESY spectrum. **(D)** HSQC spectrum.

TABLE S1 Recipes for liquid screening media. Quantities of components are given in g/L. SM = screening media.

	Maltodextrin	Bacto Soy peptone	Bacto Peptone	$CaCl_2$ [0.02 M]	$CaCl_2*2H_2O$	$CaCO_3$	Casamino acids	Corn steep liquor	EDTA	Glucose	Glycerol solution	K_2HPO_4	L-Glutamate	L-Proline	Malt extract	Maltose	Meat Extract	$MgSO_4$ [0.2 M]	$MgSO_4*7H_2O$	Molasses (cane)	MOPS	NaCl	Oatflour	Cotton seed flour	Soluble starch	Soya flour	Tomato paste	Trace salts No.1	Yeast extract	$ZnSO_4*7H20$	Distilled water (to 1L)	Tap water (to 1L)	pH
	g	g	g	mL	g	g	g	g	g	g	g	g	g	g	g	g	g	mL	g	g	g	g	g	g	g	g	g	mL	g	g			
**SM3**	**50.0**					**2.5**				**5.0**		**0.3**								**3.0**						**25.0**					**x**		**7.0**
**SM5**			**20.0**			**0.4**				**15.0**	**10.0**						**8.0**														**x**		**7.2**
**SM6**	**20.0**							**40.0**										**0.5**				**2.5**										**x**	**7.0**
**SM7**				**10.0**							**20.0**	**2.0**	**1.5**	**15.0**				**10.0**			**20.9**	**0.5**						**5.0**			**x**		**6.5**
**SM12**			**4.0**			**5.0**				**50.0**							**4.0**					**2.5**				**10.0**						**x**	**7.6**
**SM14**		**20.0**								**10.0**							**5.0**					**5.0**								**0.01**	**x**		**7.0**
**SM15**					**0.029**		**11.5**		**0.25**		**23.0**	**0.5**							**0.49**		**20.9**	**0.5**						**5.0**			**x**		**6.5**
**SM18**						**8.0**				**15.0**										**20.0**				**25.0**	**40.0**							**x**	**7.2**
**SM19**										**2.0**													**15.0**				**40.0**					**x**	**6.0**
**SM20**			**5.0**													**20.0**	**5.0**		**1.0**			**3.0**							**3.0**			**x**	**7.2**
**SM25**			**10.0**								**40.0**				**21.0**																**x**		**6.3**
**SM30**										**2.0**													**15.0**				**40.0**					**x**	**4.5**
**SM32**			**10.0**								**40.0**				**21.0**																**x**		**4.5**

TABLE S2 Putative NMR assignments. ND = not determined.

Amino Acid	Position	δH	δC	Amino Acid	Position	δH	δC
1-Cys	NH_2	ND	-	12-Phe	NH	ND	-
	α	ND	ND		α	ND	ND
	β	ND	ND		β	ND	ND
2-Ala	NH	8.00	-		γ	-	ND
	α	4.24	48.6		δ	7.17	129.3
	β	1.19	20.9		ε<br>ϕ	7.21	128.6<br>127.0
3-Ser	NH	8.54	-	13-Ala	NH	7.42	-
	α	5.22	54.3		α	4.64	54.1
	β	3.76	ND		β	1.19	18.6
4-S-Abu	NH	7.83	-	14-Cys	NH	8.80	-
	α	4.38	60.8		α	3.24	56.9
	β	3.29	48.0		β	2.74<br>2.64	35.7
	γ	1.11	19.1				
5-Cys	NH	9.02	-	15-HO-Asp	NH	7.17	-
	α	4.30	55.4		α	4.53	58.6
	β	2.60<br>2.30	38.5		β	4.30	72.4
					OH	3.82	-
6-N-Ala	NH	7.81	-	16-Gly	NH	7.48	-
	α	4.95	55.8		α	4.42<br>4.65	58.7
	β	3.76<br>3.68	64.4	17-Ser	NH	8.36	-
7-Ala	NH	ND	-		α	5.00	53.8
	α	ND	ND		β	3.52	70.3
					OH	5.33	-
	β	1.18	19.4	18-S-Abu	NH	8.47	-
8-Gly	NH	ND	-		α	3.62	56.2
	α	ND	ND		β	1.85	24.9
9-Pro	α	3.98	61.4		γ	ND	ND
	β	1.63<br>1.85	29.2	19-Lys	NH	ND	-
	γ	ND	ND		α	ND	ND
	δ	3.62	46.9		β	ND	ND
10-Phe	NH	ND	-		γ	ND	ND
	α	ND	ND		δ	ND	ND
	β	2.97	36.2		ε	ND	ND
	γ	-	ND		NH	ND	-
	δ	7.29	128.8		COOH	-	10.22
	ε<br>ϕ	7.11	129.4<br>126.7				
11-S-Abu	NH	ND	-				
	α	ND	ND				
	β	ND	ND				
	γ	ND	ND				
